# A balanced chromosomal translocation involving chromosomes 3 and 16 in a patient with Mayer-Rokitansky-Kuster-Hauser syndrome reveals new candidate genes at 3p22.3 and 16p13.3

**DOI:** 10.1186/s13039-016-0264-6

**Published:** 2016-07-30

**Authors:** Lacey S. Williams, Hyung-Goo Kim, Vera M. Kalscheuer, J. Matthew Tuck, Lynn P. Chorich, Megan E. Sullivan, Allison Falkenstrom, Richard H. Reindollar, Lawrence C. Layman

**Affiliations:** 1 Section of Reproductive Endocrinology, Infertility, & Genetics, Department of Obstetrics & Gynecology, Medical College of Georgia, Augusta University, Augusta, GA USA; 2 Research Group Development and Disease, Max Planck Institute for Molecular Genetics, Berlin, Germany; 3 American Society for Reproductive Medicine, Birmingham, AL USA; 4 Department of Neuroscience & Regenerative Medicine, Augusta University, Augusta, GA 30912 USA; 5 Department of Physiology, Medical College of Georgia, Augusta University, Augusta, GA 30912 USA

## Abstract

**Background:**

Mayer-Rokitansky-Kuster-Hauser (MRKH) syndrome, or the congenital absence of uterus and vagina, is the most severe anomaly of the female reproductive tract. It affects 1 in 5,000 females, and is the second most common cause of primary amenorrhea. The etiology remains unknown in most patients, although four single gene defects and some repetitive copy number variants (CNVs) have been identified. Translocations in MRKH patients are very rare, and reported only in three patients previously without breakpoint mapping. We have identified the fourth MRKH translocation patient and are the first to characterize the breakpoints mapped by molecular methods.

**Results:**

The proband is a 17- year old white female with agenesis of the uterus and vagina who had a peripheral blood karyotype revealing a de novo balanced translocation 46,XX,t(3;16)(p22.3;p13.3)dn. There were no known related anomalies present in the proband or her family. No CNVs were found by chromosomal microarray analysis, and no genes were directly disrupted by the translocation. DNA sequencing of six nearby candidate genes—*TRIM71, CNOT10, ZNF200, OR1F1*, *ZNF205*, and *ZNF213*—did not reveal any mutations. RT-qPCR of proband lymphoblast RNA for 20 genes near the breakpoints of 3p22.3 and 16p13.3 showed significantly altered expression levels for four genes in the proband compared to three white female controls, after correction for multiple comparisons. Reduced expression was seen for *CMTM7* and *CCR4* on 3p22.3, while increased expression was observed for *IL32* and *MEFV* on 16p13.3.

**Conclusion:**

We have mapped the breakpoints of our t(3;16)(p22.3;p13.3) translocation patient using molecular methods to within 13.6 kb at 3p22.3 and within 1.9 kb for 16p13.3 and have suggested 10 nearby genes that become plausible candidate genes for future study.

## Background

Abnormal development of the uterus and vagina affects 7-10 % of women, comprising a significant cause of impaired reproductive function [1]. Mayer-Rokitansky-Kuster-Hauser (MRKH) syndrome, also known as congenital absence of the uterus and vagina or mullerian aplasia, is the most severe anomaly of the female reproductive tract in which the uterus and vagina are absent from birth [1]. MRKH (the name patients prefer) affects approximately 1 in 5,000 females, and is the second most common cause of primary amenorrhea [1]. These women are 46,XX females that lack the vagina and most of the uterus, although fallopian tubes may be present [2]. Ovaries are present with normal function, thus patients undergo spontaneous puberty.

MRKH is commonly classified with regard to the presence or absence of additional anomalies [2, 3]. Isolated agenesis of the uterus and vagina occurs in about two-thirds of MRKH patients, classified as Type I. The remaining one-third of MRKH patients have one or more associated anomalies, and are classified as Type II. More frequent associated anomalies involve the kidneys with renal agenesis (32 %) and the skeletal system (12 %) [3]. Less commonly, women with MRKH may also present with deafness, inguinal hernia, or abnormalities of the cardiac or nervous systems [3].

While the etiology of MRKH is not well understood, disease clustering in >67 families clearly indicates a genetic component [4]. A number of candidate genes including *AMH, AMHR, WT1*, *WNT7A*, *CFTR*, *GALT*, *HOXA7*, *HOXA13, PBX1, HOXA10, RARG, RXRA, CTNNB1, PAX2, LAMC1, DLGH1*, and *SHOX* have been screened for mutations in small numbers of MRKH patients, but no mutations were found [5–9]. Genomic regions 16p11 and 17q12 most commonly have been found to have copy number variants (CNVs) identified by chromosomal microarray analysis implicated in MRKH, but causation is difficult to prove [10, 11]. It is currently not clear if MRKH is a genomic disorder or if a single gene or several genes within these CNVs could be etiologic [5]. Single gene defects have uncommonly been identified–only a few patients have *WNT4* [12–15], *LHX1* [16], *HNF1B* [17], or *TBX6* [18] gene mutations. The molecular basis for MRKH remains unknown in the vast majority of patients [5].

Patients with balanced translocations provide a unique and valuable opportunity to identify genes involved in human genetic disorders [19]. The derivative chromosome breakpoint may disrupt or dysregulate genes, suggesting a genomic region of etiologic candidate genes [20]. This method has been successful in identifying candidate genes in other disorders, and may be valuable to elucidating the molecular mechanisms of MRKH [20]. Only three MRKH patients with balanced translocations have been reported in the literature, but fine mapping by molecular methods has not been performed for any of them [21, 22]. In this study we present an MRKH patient with a de novo balanced translocation of 46,XX,t(3;16)(p22.3;p13.3)dn with the purpose to: 1) identify the molecular breakpoints of 3p22 and 16p13; 2) propose candidate genes for MRKH; and 3) compare the proband to other MRKH patients with balanced translocations presented in the literature.

### Case presentation

**Fig. 1:**
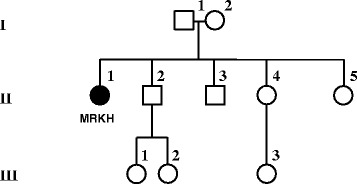
The pedigree is shown for the proband with a de novo t(3;16) translocation

The proband is a 17-year-old white female with agenesis of the uterus and vagina who had a peripheral blood karyotype revealing a de novo balanced translocation 46,XX,t(3;16)(p22.3;p13.3). She has no associated renal, skeletal, or hearing anomalies. She has two unaffected sisters and two brothers (Fig. 1). Both parents and her unaffected sister II-5 have normal karyotypes, and all three nieces (III-1, III-2, and III-3) have no known mullerian, renal, or skeletal defects.

## Results

**Fig. 2:**
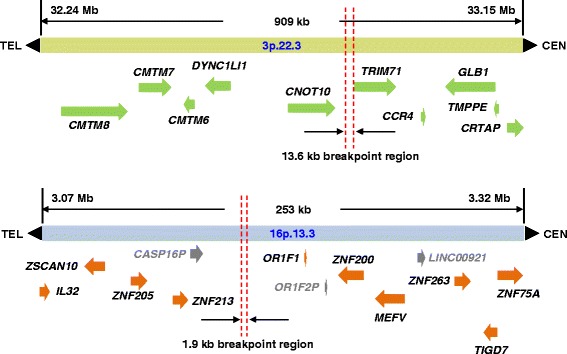
Shown are the breakpoints with nearby candidate genes for chromosome 3p22.3 (top) and 16p13.3 (bottom)

Flow sorting of both derivative chromosomes 3 and 16 followed by comparative genomic hybridization (CGH) showed no deletions or duplications, and the breakpoints were localized (Fig. 2). The breakpoint of der(3) was narrowed to within 13.6 kb at 3p22.3; and to within 1.9 kb on 16p13.3. In neither derivative chromosome was a gene directly disrupted, but nearby genes become candidate genes for MRKH (Fig. 2).

Six genes near the breakpoints were prioritized as reasonable candidate genes based upon either expression in appropriate tissues and/or proposed gene function. DNA sequencing was performed on genomic DNA from 51 MRKH patients for the protein coding exons and splice junctions for the two closest candidate genes near the breakpoint at 3p22.3---*TRIM71* centromeric and *CNOT10* telomeric. In addition, *ZNF200*, *OR1F1, ZNF213*, and *ZNF205* at 16p13.3 were sequenced in 27 MRKH patients. No likely causative (nonsense, frameshift, or splice site) or potentially causative (nonsynonymous missense changes) variants were identified for any of these six genes.

**Fig. 3:**
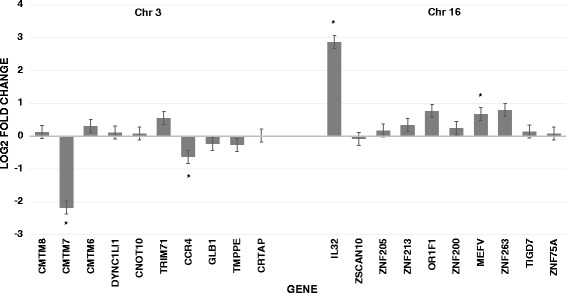
Log2 fold-change from RT-qPCR of 20 genes located near the breakpoints of chromosome 3p22.3 and 16p13.3. Statistically significant altered gene expression (*P* < 0.00001) is indicated by an asterisk for four genes

Since the translocation could disrupt regulatory elements, we performed RT-qPCR from lymphoblastoid RNA from the proband for 20 genes near the breakpoints to see which ones, if any, had altered expression. This could provide additional supportive evidence for a candidate gene(s). Using the CT method [23], four genes in the proband had significantly different expression from the mean of three white female controls after correction for multiple comparisons. These included two genes on chromosome 3p22.3—*CMTM7* with 0.22 fold (78 % reduction) and *CCR4* with a 0.64 fold (36 % reduction) change—and two genes on chromosome 16p13.3—*IL32* with 7.3 fold and *MEFV* with 1.6 fold increases in expression (Fig. 3). The altered expression of these four genes reached significance by a Z-test with a *P* < 0.00001 (*P* < 0.0025 was considered significant after Bonferroni correction).

## Discussion and conclusions

The etiology of MRKH remains largely unknown [5, 24], although when families are examined, autosomal dominant or multifactorial/polygenic inheritance seems most likely [4]. Several potentially causative repetitive CNVs have been described—most commonly deletions in 17q12 (n > 9) and 16p11 (n >5) [5], but it is not clear if they play a pathogenic role [5, 24]. Single gene defects are found even less commonly. To date, four *WNT4* [12–15], one *LHX1* [16], one *HNF1B* [17], and several *TBX6* [18] mutations and intragenic CNVs [18] have been characterized, indicating that many more genes for MRKH remain to be discovered.

While syndromic families have been reported, they are few and small in size [4]. Thus their ability to show genetic segregation among affected family members is very limited. Most MRKH probands do not have a family history of other affected individuals. The inability of affected women to conceive and carry children is a significant barrier to characterizing inheritance patterns and identifying causative genes. However, when MRKH patients who underwent surrogacy were surveyed by a questionnaire, 32/53 (60 %) responded and 34 liveborns were delivered. Half were females, and only one child—a male—had a middle ear defect and hearing loss (1 of 17 = 5.9 %), consistent with an associated phenotype of MRKH [25]. It is possible that less severe associated anomalies may not have been ascertained.

Genomic rearrangements, which include balanced translocations, occur more frequently than de novo point mutations, and may directly disrupt a gene, thereby altering its normal function, or result in a sufficient “position effect” to alter or impair its regulatory mechanisms. [20] Candidate genes suggested by this position effect have facilitated successful identification of causative mutations in multiple disorders, including *PAX6* in aniridia, *PITX2* in Reiger syndrome, *FOXL2* in blepharophimosis/ptosis-epicanthus inversus syndrome, *SOX9* in campomelic dysplasia, *SRY* in sex reversal, *SIX3* in holoprosencephaly, and *WDR11* in hypogonadotropic hypogonadism/Kallmann syndrome [20, 26]. Balanced translocations in affected individuals highlight a narrow section of the genome that is disrupted, and can provided much needed clues to the etiology of human disease [27].

In MRKH, only several translocations have been reported in the literature (Table 1). In 1988, Kucheria et al. [21] reported two unrelated females with MRKH who had translocations involving chromosomes 12q and 14q (no further cytogenetic details are provided). In 1999, Amesse et al. [22] reported an adolescent with MRKH, who also lacked nipples and breasts (amastia/amelia), with associated urinary reflux, urinary incontinence, and megaurethra. In peripheral blood, she had a de novo 46,XX,t(8;13)(q22.1;q32.1). When buttock fibroblasts were analyzed by karyotype, a 4:1 chromosomal mosaicism was observed, with the predominant 46,XX,t(8;13)(q22.1;q32.1) cell line, but also a more complex t(1;12)(q23.1;q24.3),t(6;6)(q15;p25) translocation. Amesse et al. [22] acknowledge that the patient’s lack of breast and areolar tissue is uncommon in MRKH, and that the translocation of the less frequent cell line is of unknown significance. These two reports were prior to the advent of chromosomal microarrays, and so the molecular breakpoints were not analyzed to determine the specific breakpoint. Our patient is the first MRKH translocation patient, with a typical clinical presentation, to have the translocation breakpoints mapped by molecular methods, which should help narrow down the number of putative candidate genes.

**Table 1:** Reported translocations in patients with MRKH

Author	Case #	Translocation	De novo	Ethnicity	Phenotype
Kucheria et al. [21]	1	46,XX,t(12q;14q)	?	Not stated	MRKH
	2	46,XX,t(12q;14q)	?	Not stated	MRKH; renal agenesis
Amesse et al. [22]	3	46,XXt(8;13)(q22.1;q32.1) in blood; Mosaic in 4:1 ratio in buttock fibroblasts for minor cell line of 46,XX, t(1;12)(q23.1;q24.3),t(6;6)(q15;p25)	Yes	Not stated	MRKH; congenital amastia/amelia,urinary reflux; urinary incontinence, megarethra
Williams et al.; Current case	4	46,XX, t(3;16)(p22.3;p13.3).	Yes	Caucasian Northern European	MRKH

In the current study, we mapped the molecular breakpoints in an MRKH female with a de novo 46,XX,t(3;16)(p22.3;p13.3)dn. A chromosomal microarray was used to narrow down the breakpoints of both derivative chromosomes to within 13.6 kb on der(3) and within 1.9 kb on der(16). No deletions/insertions were identified, which could have been potential confounders in this patient. No gene was directly disrupted, but the genes closest on either side of the breakpoint became prime positional candidate genes. We did not amplify and clone the breakpoints in this patient since the breakpoint region is not contained within a structural gene. *TRIM71* (tripartite motif-containing 71) on the centromeric side of the breakpoint (Fig. 2) has been reported to be involved in the timing of organ formation during development [28], while *CNOT10* on the telomeric side of the breakpoint is involved in transcription [29]. We did not identify mutations in our available sample of 51 MRKH patients in these genes, which suggests they are not a common etiologic factor in MRKH. However, we cannot exclude that genes with mutation frequencies of 1-2 % could occur. We also performed DNA sequencing for three genes near the breakpoint on chromosome 16p13.3, namely *OR1F1* on the centromeric side and three zinc finger genes involved in transcription*—ZNF213* and *ZNF205* on the telomeric side and *ZNF200* on the centromeric side of the breakpoint. In 27 unrelated MRKH patients, no putative mutations were identified.

In addition, RNA was extracted from peripheral lymphoblastoid cells from our proband, and 20 genes near the breakpoints of both derivative chromosomes were subjected to RT-qPCR. Expression was normalized to the *GAPDH* reference gene and compared to three white female controls. Four genes were found to have statistically altered gene expression after correction for multiple comparisons. It is interesting that both genes on chromosome 3p22.3, *CMTM7* and *CCR4*, have reduced expression, while both genes on 16p13.3, *IL32* and *MEFV*, show increased expression. It is interesting to speculate whether the 3p genes regulate the 16p genes or vice versa. This will have to be determined experimentally in future studies. Two genes—*CMTM7* and *IL32*—have the most profound differences, so they are the most plausible involved genes within the breakpoints.

Through the fine mapping of the breakpoints in this translocation patient, we have identified six candidate genes that require future testing based upon the location and proposed role in mullerian development—*TRIM71* and *CNOT10* on chromosome 3, and *OR1F1, ZNF213*, *ZNF200*, and *ZNF205* on chromosome 16. By RT-qPCR, we show altered expression of four additional genes—*CMTM7* and *CCR4* on chromosome 3, and *IL32* and *MEFV* on chromosome 16—that become reasonable candidate genes. Balanced chromosomal translocations may exert a position effect on genes within 10 kb or even up to a megabase away from the breakpoint [20]. However, additional patients will need to be collected and studied to adequately test the hypothesis that these are involved in the pathogenesis of MRKH. Since deleterious gene mutations affecting most reproductive disorders usually occur at a frequency between 1-5 % [30, 31], large cohorts are necessary for sufficient power to identify new causative genes.

To date, balanced translocations from MRKH patients have not yielded causative genes in the etiology of the syndrome, but our more comprehensive molecular analysis of a t(3;16) translocation pinpoints specific chromosomal regions that will require further study. It is also certainly possible that causal variants could be localized outside the structural genes and might be detected by whole genome sequencing approaches. Nevertheless, the complicated genetic basis of MRKH remains unsolved, but study of large cohorts and families by innovative molecular approaches, appropriate in vitro confirmation of identified variants, and relevant mouse models will be necessary.

## Methods

### MRKH Patients

Our 52 patients with MRKH, except the proband with the t(3;16), were 46,XX females with adult (Tanner 5) breast development, normal pubic hair, and an absent vagina. Ultrasound or MRI was performed on some patients revealing hypoplasia or aplasia of the uterus. Associated anomalies were present in patients as follows: unilateral renal agenesis (*n* =7), skeletal anomalies (*n* = 6), cardiac defects (*n* = 3), and hearing loss (*n* = 5). All patients consented to have blood drawn for molecular analysis and signed a consent approved by the Human Assurance Committee of the Medical College of Georgia at Augusta University. LCL was funded by NIH HD33004 and the Department of Ob/Gyn at Augusta University, which were the sole sources of funding.

### CGH arrays

For breakpoint mapping, we did array painting essentially as described previously [32]. Briefly, the EBV-transformed patient lymphoblastoid cell line was treated with colcemid, and metaphase chromosomes were then isolated and flow-sorted. Approximately 6,000 flow-sorted chromosomes were used directly for amplification with the GenomiPhi V2 DNA Amplification Kit (GE Healthcare, Piscataway, NJ, USA) according to the manufacturer’s recommendations. One microgram of amplified DNA from each derivative chromosome was labelled via Agilent´s Genomic DNA Enzymatic Labeling Kit Plus (Agilent). To each labelling reaction 100 ng of genomic control DNA were added to ensure proper placement of the grid for subsequent image analysis. These probes were hybridized to a custom array for high resolution breakpoint mapping. All hybridizations were done according to the manufacturer's recommendations for array CGH experiments (Agilent). Further analysis and visualization of array painting data was done using the array CGH software package CGHPRO [33].

### Sanger DNA sequencing

DNA sequencing of all protein coding exons and splice junctions was performed on 51 MRKH patients for 4 exons in *TRIM71* (NM_001039111.2) and 19 exons in *CNOT10* (NM_015442.2) at the 3p22.3 breakpoint. In addition, 27 MRKH patient DNAs were subjected to Sanger sequencing for the 4 exons of *ZNF200* (NM_003454.3), 1 exon of *OR1F1* (NM_012360.1)*,* 5 exons of *ZNF213* (NM_004220.2), and 6 exons of *ZNF205* (NM_001278158.1) at 16p13.3.

### RT-qPCR methods

RNA was extracted from lymphoblastoid cells of the proband with the translocation and three white female controls using the TRI REAGENT (Molecular Research Center, Inc, Cincinnati, OH) standard protocol. RT-qPCR primers were designed with a product size of <155 bp for 20 genes around the chromosome 3 and chromosoms16 breakpoints. These genes included: *CMTM6, CMTM7, CMTM8, DYNC1LI1, CNOT10, TRIM71, CCR4, GLB1, TMPPE, CRTAP, IL32, ZSCAN10, ZNF205, ZNF213, OR1F1, ZNF200, MEFV, ZNF263, TIGD7*, and *ZNF75A*. *GAPDH* was used as the internal reference control.

The RT reaction was performed using the ABI-High Capacity cDNA Reverse Transcription Kit [RXN w/o RNAse Inhibitor] (Thermo Fisher Scientific, Waltham, MA). 2 μg of RNA were used in the 20 μl reaction with RT conditions of: 25 °C for 10 min, 37 °C for 120 min, 85 °C for 5 min, 4 °C cooling. The cDNA product was diluted 1:5 for the qPCR reaction, which was run on a Roche LightCycler 96 using the FastStart Essential DNA Green Master Kit protocol (Roche Diagnostics Corporation, Indianapolis, IN) with SYBR green. In the 25 μl reaction volume 2 μl of cDNA was used and each primer concentration was 25 pmol. *GAPDH* and each of the test genes were run in duplicate/run. Three separate experiments were performed. LightCycler 96 settings were: pre-incubation at 95 °C for 10 min, 3-step amplification (45 cycles of 95 °C 10 s, 60 °C 10 s, 72 °C 10 s), melting (95 °C 10 s, 65 °C 60 s, and 97 °C 1 s), and a final cooling at 37 °C for 30 s. C_T_ values [23] and standard deviations were calculated and statistics were done using Z Score values with a Bonferroni correction. With the Bonferroni correction for 20 genes, a *P* <0.0025 was considered significant.

## Abbreviations

μg, microgram; μl, microliter; cDNA, complementary DNA; CGH, comparative genomic hybridization; CNVs, copy number variants; der(16), derivative chromosome 16; der(3), derivative chromosome 3; DNA, deoxyribonucleic acid; EBV, Epstein Barr virus; kb, kilobase; min, minute; MRKH, Mayer-Rokitansky-Kuster-Hauser syndrome; pmol, picomole; qPCR, quantitative polymerase chain reaction; RNA, ribonucleic acid; RT-qPCR, real time-quantitative polymerase chain reaction; t(3;16), translocation of chromosomes 3 and 16
